# Spontaneously spotting and applying shortcuts in arithmetic—a primary school perspective on expertise

**DOI:** 10.3389/fpsyg.2014.00556

**Published:** 2014-06-10

**Authors:** Claudia Godau, Hilde Haider, Sonja Hansen, Torsten Schubert, Peter A. Frensch, Robert Gaschler

**Affiliations:** ^1^Department of Psychology, Humboldt-Universität zu BerlinBerlin, Germany; ^2^Cluster of Excellence: Image Knowledge Gestaltung, an Interdisciplinary LaboratoryBerlin, Germany; ^3^Department of Psychology, Universität KölnKöln, Germany; ^4^Department of Psychology, Universität Koblenz-LandauLandau, Germany

**Keywords:** expertise, numerical cognition, arithmetic, commutativity, spontaneous strategy application

## Abstract

One crucial feature of expertise is the ability to spontaneously recognize where and when knowledge can be applied to simplify task processing. Mental arithmetic is one domain in which people should start to develop such expert knowledge in primary school by integrating conceptual knowledge about mathematical principles and procedural knowledge about shortcuts. If successful, knowledge integration should lead to transfer between procedurally different shortcuts that are based on the same mathematical principle and therefore likely are both associated to the respective conceptual knowledge. Taking commutativity principle as a model case, we tested this conjecture in two experiments with primary school children. In Experiment 1, we obtained eye tracking data suggesting that students indeed engaged in search processes when confronted with mental arithmetic problems to which a formerly feasible shortcut no longer applied. In Experiment 2, children who were first provided material allowing for one commutativity-based shortcut later profited from material allowing for a different shortcut based on the same principle. This was not the case for a control group, who had first worked on material that allowed for a shortcut not based on commutativity. The results suggest that spontaneous shortcut usage triggers knowledge about different shortcuts based on the same principle. This is in line with the notion of adaptive expertise linking conceptual and procedural knowledge.

## Introduction

Expertise has various manifestations and could be defined as consistently superior performance within a specific domain relative to novices and relative to other domains (Ericsson and Lehmann, 1996). The development of expertise in real-world domains involves a complex interplay of changes in perception, categorization, memory, problem solving, coordination, skilled action, and other components of human cognition (Palmeri et al., 2004). Expert's flexibility has been frequently discussed and there exist two contradictory perspectives. Research on creativity and skill acquisition has been used to illustrate that more knowledge can make one less flexible (i.e., Luchins, 1942; Logan, 1988). However, research on expertise suggested that experts are more flexible and creative in their thought patterns (see summary in Bilalić et al., 2008a). Both options might be possible depending on the expertise level and the problem difficulty. Investigating chess experts Bilalić et al. (2008a) found that “super experts” were flexible and find the optimal solution first or at least find it quickly after perceiving a salient but non-optimal solution.

Here, we focus in the domain of mathematics on spontaneously spotting and applying shortcuts in arithmetic and whether with further experience students become increasingly able to generate rapid adequate actions with less and less effort (Ericsson, 2008). Mathematic students used significantly larger numbers of appropriate strategies than adults with less expertise (Dowker et al., 1996). Experts have to be able to recognize spontaneously and without instruction that a specific element of their knowledgebase can be applied in a specific situation. It would not suffice if they possessed elaborate conceptual knowledge as well as procedures to apply it, but needed to wait for someone to tell them that the knowledge can be applied in the given situation. This someone would rarely drop by.

In recent years, research in primary school arithmetic has started to tackle this issue for a domain in which everyone should acquire elaborate knowledge. Learning about mathematical principles and procedures should lead to knowledge that can be applied across a wide range of situations (e.g., Hatano and Oura, 2003). Given the role of self-guided learning and performance in the development of mathematical abilities and concepts, recent studies have focused on the question how and when children spontaneously recognize that an everyday situation can be tackled by mathematical thinking (Hannula and Lehtinen, 2005; Hannula et al., 2010; McMullen et al., 2011). Furthermore, children should develop the skills necessary to flexibly spot and apply shortcut strategies spontaneously. It is not sufficient if they can apply a shortcut when explicitly told to do so. Adaptive expertise (Verschaffel et al., 2009) includes to autonomously regulate whether (a) to solve an arithmetic problem in a standard way or to (b) search for / apply a shortcut.

Taking the commutativity principle as a model case, past research has explored how children spontaneously spot and apply shortcuts that allow saving effort in addition problems by flexibly changing the order of addends. Wealth of research has shown that children have at least some understanding of the concept of commutativity before entering school (Baroody and Gannon, 1984; Resnick, 1992; Cowan and Renton, 1996; Wilkins et al., 2001; Canobi et al., 2003). After interviewing elementary school children how they solved problems with two addends, (Baroody et al., 1983) report an extensive use of commutativity. During development children increasingly integrate conceptual knowledge about mathematical principles and procedural knowledge about shortcuts (Haider et al., 2014). Knowledge integration should lead to transfer between procedurally different shortcuts that are based on the same mathematical principle and therefore likely both associated to the respective conceptual knowledge. In a first step, (Gaschler et al., 2013) provided a correlative study to explore this idea. They assessed spontaneous usage of two procedurally different shortcuts that are both based on the commutativity principle in children of different age. While shortcut usage was observed from second grade onwards, correlations between the usage of the two different shortcuts only emerged by grade four. In the current study we aimed at moving beyond correlational data. We tested whether being exposed to one commutativity-based shortcut helps to spot and apply a different shortcut option based on the same mathematical principle. Note that in a parallel line of research, we have observed that instructions do not seem to do the job. Instructing children to use one specific shortcut does hinder rather than assist them in spontaneously spotting and applying a different shortcut based on the same mathematical principle later on (Godau et al., submitted). Instructions about specific procedures might corrupt flexibility in shortcut usage (cf. ErEl and Meiran, 2011). Even when participants knew that a formerly instructed rule would no longer apply, they found it difficult to search for different shortcut options (see also Bilalić et al., 2008a,b; Bilalić and McLeod, 2014). Therefore, in the current work we focused on spontaneous use of the strategies. We explored whether it is possible to foster the discovery and application of shortcut strategies by transfer between different non-instructed shortcut strategies that are based on the same mathematical principle. Note that according to Baroody and Gannon (1984) understanding of commutativity was not evident in all those who invented shortcuts, but in all those who comprehend addition as a binary rather than as a unary operation. The unary view would suggest that one number is added to another, rather than that they are added together.

Specifically, the commutativity principle enables students to flexibly change the order of addends within a problem. For instance, given the problem 4 + 7 + 6, it might be easier to calculate (6 + 4) + 7 (6 + 4 adds up to 10 which makes it easy to finally add 7, i.e., “Ten-strategy”). One can also use commutativity across problems. If, for instance, a student receives the problem 8 + 5 + 7 =?, and then 5 + 7 + 8 =?, he/she can refrain from calculating the second problem presupposed he / she recognizes the applicability of the commutativity principle (i.e., “addends-compare strategy”). Three-addends problems were used, because we wanted to investigate usage of the commutativity principle with unfamiliar problems. It is debatable if three-addends problems imply only the commutativity principle or additionally also the associativity principle. Associativity is the property that problems in which terms are decomposed, and recombined in different ways, have the same answer [(a + b) + c = a + (b + c)]. In the problems we used, children have to change the order of the addends [a + b + c = (a + c) + b], because otherwise it is not possible to add a + c first. Commutativity justifies changing the order or sequence of the operands within an expression while associativity does not.

In Experiment 1, we used eye tracking to explore how children search and apply different commutativity-based shortcuts. Verschaffel et al. (1994) presented third-graders with three-addends problems and assessed eye movements combined with verbal report and found that in 71% of all possible cases commutativity was used. We used a different approach, as we rather were interested in whether children spontaneously start search processes when, after a change in the material one shortcut option is no longer present. The findings suggested that being offered an opportunity to apply one commutativity-based shortcut can help to search for and apply a different shortcut based on the same principle when the first one is no longer feasible. In Experiment 2, we explored whether transfer from shortcut to shortcut might be concept specific: on the one hand, it seems plausible that shortcuts based on the same mathematical principle trigger each other because they are linked to one-another directly or indirectly (as they are both linked to the common conceptual knowledge). This perspective is in line with research suggesting that mathematical knowledge develops in an iterative fashion, with conceptual change influencing procedural change and vice versa (Byrnes and Wasik, 1991; Hiebert and Wearne, 1996; Rittle-Johnson et al., 2001; Waldmann, 2006). For instance, Canobi (2009) showed that children's conceptual advances were predicted by their initial procedural skills. On the other hand, transfer from shortcut to shortcut might occur place for motivational reasons unrelated to the specific shortcut and underlying mathematical principle. After having experienced that task processing can be simplified by a shortcut, one might be more apt to search for and apply *any* shortcut, as one has learned that attractive shortcut options do seem to exist in the material provided.

## Experiment 1

In Experiment 1, we used eye tracking in order to explore the fixation patterns reflecting the usage of shortcut strategies. We were furthermore interested in how fixation patterns reflect how people accommodate to being presented with new sets of arithmetic problems within which the previously feasible shortcut no longer applies (but instead a different shortcut). To this end, children at first had to solve problems that could be facilitated by the ten-strategy (of three addends, the first and the last add up to 10). After that, they were presented with problems that allowed for the use of the addends-compare strategy (some problems contained the same addends as their precursor in different order). Both strategies are based on the commutativity principle.

### Method experiment 1

#### Participants

Twenty children participated in Experiment 1 (mean age 8.6 years). They were tested individually in a laboratory at Humboldt-Universität, Berlin.

#### Procedure and materials

Research procedures of these experiments were approved in a peer review process for applying for public funding (German Research Foundation, DFG) and were completed with approval of the Institutional Review Board of the Department of Psychology at Humboldt-Universität, Berlin. Students were informed about the content of the study and that data analysis would preserve anonymity. We ensured written informed consent of the parents. Children were than tested individually with a 250 Hz video-based eye tracker (SMI RED 250). Packages of six problems in black on a gray background were shown on a 22 TFT monitor, with the student sitting at approximately 50 cm distance. Digits were approximately 0.5 cm wide and 1 cm tall.

Children started with a five-point calibration. Afterwards the experimenter showed a single example problem and explained that the children should utter the result as quickly and as accurately as possible. Children started the main part by working on two screens with six ten-strategy problems each (first and last addend add up to 10). They then completed two screens with addends-compare problems intermixed with baseline problems. Two of six problems per screen contained identical addends in different order as the preceding problem (problems listed in the Supplementary materials). Each problem was presented in one line and consisted of three different addends between 2 and 9 (maximum result was 24; 0 and 1 were excluded as addends). We balanced problem size between the addends-compare problems and the baseline problems so that they were equally difficult for children unless they used the shortcut (for more details Gaschler et al., 2013; Haider et al., 2014).

Children were presented the first screen (of two) with six ten-strategy problems. The experimenter moved the cursor to the right of the equal sign of the first problem and waited for an answer. The answer was immediately entered as the time log of the first key press served to determine the calculation time as the span from the cursor allocation to the first (i.e., two-digit results) key press of entering the result for the current problem. After entering the answer, the experimenter moved the cursor to the next problem. The entered results remained visible on the screen while working on the remaining of the six problems of the package. This was especially important for the work on the two screens with addends-compare problems later on. If they had spotted that the addends of a problem were the commuted version of the preceding problem, that way they were provided with the opportunity to access the solution they had given on the previous problem.

### Results

**Figure 1:**
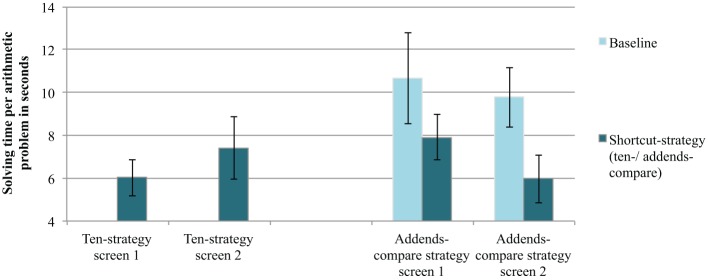
**Mean calculation time per arithmetic problem in Experiment 1**. Error bars indicate the standard error of the mean.

The computerized assessment allowed to track solution times on the level of single problems. As previously mentioned, students calculated 12 ten-strategy problems (Screen 1 and 2) and afterwards worked on yet another 12 problems, four of them allowed for the addends-compare strategy (Screen 3 and 4). Figure 1 shows the mean solution times per problem for each screen. Students were faster on addends-compare problems as compared to baseline problems. A 2 (screen: first vs. second) × 2 (problem type: addends-compare problem vs. baseline problem) ANOVA with solution times as dependent variable revealed a significant main effect of problem type, [F_(1, 19)_ = 7.46, *p* = 0.01, η^2^_p_ = 0.28]. Neither the main effect of screen, [F_(1, 19)_ = 1.67, *p* = 0.21, η^2^_p_ = 0.08], nor the interaction effect were significant, [F_(1, 19)_ = 0.72, *p* = 0.41, η^2^_p_ = 0.04]. We did not find significant effects when repeating the above analyses with error rate as dependent variable (see Supplementary materials).

**Figure 2:**
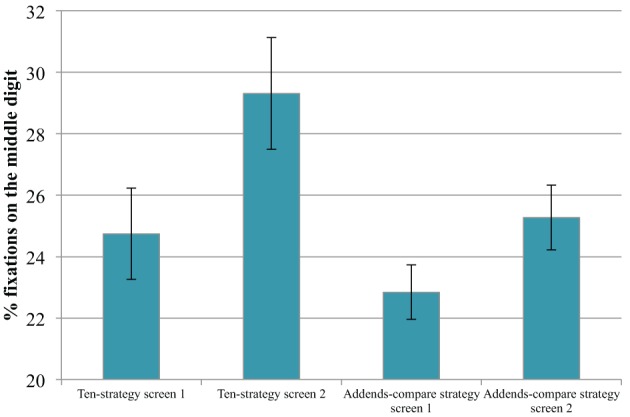
**Percent fixation frequency on second addend for ten-strategy and addends-compare strategy booklets**. The error bar displays the standard error of the mean.

The analysis of the eye tracking data suggests that the ten-strategy and the addends-compare strategy can be identified by specific fixation patterns. Using the ten-strategy, adding the first and last addend first to receive the result ten, should be fast and necessitates little fixation time on the outer numbers. Adding the middle number afterwards and uttering the result might therefore result in more fixation time on the middle number relative to the other numbers. Figure 2 suggests that the percent fixations falling on the middle vs. outer numbers of the three-addends problems are distributed in line with this reasoning. The percentage of fixations on the middle number increased from the first to the second screen of the ten-strategy problems, as students presumably discovered the structure of the problems. When the ten-strategy could no longer be used (first screen with addends-compare problems), the percent fixations on the middle digit were low again. Surprisingly, it increased on the second screen with addends-compare problems. A 2 (screen: first vs. second) × 2 (ten-strategy problems vs. addends-compare problems) ANOVA with percentage of fixations falling on the middle number as dependent variable revealed a significant main effect for strategy [F_(1, 17)_ = 6.02, *p* < 0.05, η^2^_p_ = 0.26]. Children fixated the middle digit more in problems, in which the ten-strategy could be used compared to problems on the addends-compare screens. There was also a significant main effect of screen, [*F*_(1, 17)_ = 7.91, *p* = 0.01, η^2^_p_ = 0.32], but no interaction, *F* < 1.

**Figure 3:**
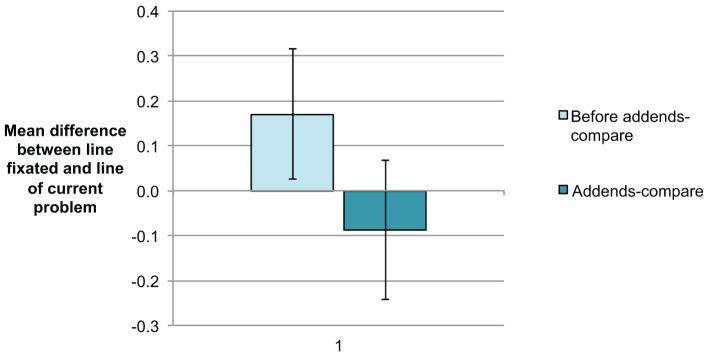
**Mean difference between current line fixated and line of current problem**. Negative values indicate fixations on preceding problems while positive values result from fixations on subsequent problems. Error bars indicate the 95% CI of the comparison of addends-compare problems vs. preceding problems.

In the ten-strategy problems, addends should be checked within a line in order to identify shortcut options. In contrast, for the addends-compare strategy, it is necessary to compare the addends between the lines. Children should thus not only fixate the addition problem they are currently solving but also the previous one or the subsequent one in order to check whether a set of addends repeats. Figure 3 presents the mean differences between (a) line fixated and (b) line of current problem. If, for instance, a student during solving a problem was fixating back on the problem in the line before, this would lead to a value of −1 for this particular fixation. While the majority of fixations were on the line of the current problem, some fixations were directed at previous (negative difference) or subsequent (positive difference) problems. We focused on comparing the above index of fixation position between the addends-compare problems and their preceding problems. Thus, addends are identical and only differ in order. We found a significant difference in the index of fixation position for these problems. In line with our assumption, students were fixating ahead on problems preceding the addends-compare problems and fixating back, once a set with identical addends was discovered, [t_(18)_ = 5.44, *p* < 0.001].

In addition to identifying eye movement patterns that are specific for the shortcuts we found a significant correlation between the increase of the fixation on the middle digit in the ten-strategy problems (Screen 2—Screen 1) and the time benefit on addends-compare strategy problems *r* = 0.49, *p* = 0.05. Thus, increased usage of the commutativity-based shortcut offered on Screen 1 and Screen 2 might help in spotting and applying the other commutativity-based shortcut offered on Screen 3 and 4.

### Discussion

Providing children with the opportunity to spontaneously (without instruction or other hints) use one commutativity-based shortcut might help them to spot and apply another shortcut based on the same mathematical principle once the first one does no longer apply. Furthermore, the eye tracking data are in line with the interpretation that search processes might start once one shortcut no longer applies. We found that children in some cases checked addends of subsequent addition problems in advance (i.e., before uttering the result to the current problem and the allocation of the cursor to the next problem). Note that this implies that the accuracy to attribute calculation time to specific arithmetic problems might be limited in setups in which multiple problems are simultaneously presented. Such arrangements resemble work on arithmetic problems on worksheets in the schooling context. Eye tracking or reliance on aggregate measures from paper-and-pencil versions might both be useful approaches to this variant of the dilemma of external vs. internal validity.

Experiment 1 provided a first hint in line with the idea that there might be transfer from one shortcut to another one. This suggests two different explanations. On the one hand, spontaneously spotting and applying shortcuts on Screen 1 and 2 might affect processing of Screens 3 and 4 on a motivational route. Participants learn that shortcut options seem to exist and can be exploited. This would suggest that such transfer could take place from any easily identifiable shortcut to a second one. On the other hand, transfer might involve specific mathematical knowledge. It might first and foremost take place between shortcuts based on the same mathematical principle. We tried to disentangle these two perspectives in Experiment 2.

## Experiment 2

This experiment focused on the question if the ten-strategy facilitated the usage of the addends-compare shortcut. For this purpose, we compared three conditions: students in the ten-strategy warm-up condition started with the ten-strategy problems followed by problems that allowed for applying the addends-compare strategy (similar to Experiment 1). In the baseline warm-up condition, children worked on material with no shortcut option at all before being transferred to the addends-compare booklet. The inversion warm-up condition started with inversion problems (e.g., 9 + 2 − 2). Thus, a shortcut *not* based on the commutativity principle was offered first. This was important in order to test whether all shortcut strategies would alter the usage of the addends-compare shortcut simply by motivation children to look for shortcuts. Alternatively, it might be that only the ten-strategy increases the probability to spot the addends-compare strategy, as it is the only shortcut strategy, which is also based on the commutativity principle. It is conceivable that offering problems with an easy-to-find shortcut option (inversion or ten-strategy) might lead students to assume that it is worthwhile to search for shortcut options in general in later material. This could accordingly lead to transfer which is simply based on the motivation to search for shortcuts. In contrast, a finding of transfer for the ten-strategy problems but not for the inversion problems would suggest that indeed triggering the basic principle of commutativity is important for transfer to occur.

### Method experiment 2

#### Participants

We tested 153 children at the end of second grade (most of them were taught in combined classes of first and second grade) and 140 children in third grade. We ensured written informed consent of the parents in collaboration with the schools. Either group was provided with advance information concerning the content of the study (calculating mental arithmetic problems) and was informed that participation was voluntary. Parents and students were also informed that data analysis would preserve anonymity. Data were acquired in a classroom setting with paper and pencil. Gender was balanced as much as possible. Eleven children (second grade) and 20 children from the third grade were excluded by median ± 3 *MAD*s. The MAD is a robust method to detect outliers by using absolute deviation from the median; for further information see (Leys et al., 2013). For the descriptive data of the sample see Table 1.

**Table 1:** **Sample data and time provided per booklet in Experiment 2**.

**Grade**	**Condition/**	**Outliers**	***N***	**Mean**	**Seconds for**
	**warm-up**		**(female)**	**age (*SD*)**	**addends-compare**
					**booklets**
2	Ten-strategy	4	48 (25)	7.1 (0.69)	240
	Baseline	1	49 (26)	7.1 (0.72)	
	Inversion	6	45 (25)	7.1 (0.62)	
3	Ten-strategy	5	41 (24)	8.0 (0.35)	180*
	Baseline	7	40 (20)	8.2 (0.71)	
	Inversion	8	39 (25)	7.8 (0.64)	

^*^We started with 210 s and than reduced it after testing one group of students in order to avoid ceiling effects.

#### Procedure and materials

The arithmetic problems were the same as in Experiment 1 and are listed in the Supplementary materials. Each problem was presented in one line and consisted of three different addends between 2 and 9 (maximum result was 24; 0 and 1 were excluded as addends). The different types of problems were presented as a paper pencil test in separate booklets. As dependent variable we measured the number of problems solved in the booklet that allowed vs. the booklet that did not allow for the addends-compare strategy. We took care that the amount of time provided per booklet was not sufficient to solve all problems so that we could use number of problems solved per time as a dependent variable (see Table 1 for time provided per booklet).

Experimental conditions differed in the warm-up booklet. The ten-strategy warm-up started with problems in which children could use the ten-strategy. The baseline warm-up conditions started with addition problems of comparable size, but that did not include any option for applying the commutativity principle to solve the problems (e.g., 4 + 3 + 5 or 7 + 6 + 2). A second control condition, the inversion warm-up condition, started with problems that allowed for a shortcut, but, importantly, not for a commutativity-based one. Inversion problems (e.g., 9 + 2 − 2) allow refraining from calculation by comparing the numbers involved in the problem mixing addition and subtraction. Thus, while the ten-strategy and addends-compare strategy are both based on the same arithmetic principle, inversion and addends-compare are not. However, on the surface the latter two shortcuts are similar as they both enables students to avoid calculation altogether (in contrast, the ten-strategy does reduce instead of avoid calculation demands).

After the warm-up phase, all children worked on five more booklets. Starting with (1) a booklet, where the addends-compare strategy could be used, they then were presented (2) a baseline booklet with no shortcut opportunities, followed by (3) another booklet, where the addends-compares strategy could be used. This second addends-compare booklet was applied as we had obtained high variability across students as well as large general practice effects in the first booklets in earlier work (Gaschler et al., 2013). Booklets 4 and 5 served the purpose to control whether the induced shortcut is known and would be used (see Table 2). The children in the ten-strategy warm-up condition received another booklet with addition problems allowing for the ten-strategy (4) plus afterwards a matched baseline booklet (5). This was also the case for children of the control condition with the baseline warm-up. The children of the inversion warm-up condition worked for the second time on a booklet with inversion problems (4) followed by a matched baseline booklet (5).

**Table 2:** **The order of the booklets in Experiment 2**.

**Condition/ Warm-up**	**Booklet 1**	**Booklet 2**	**Booklet 3**	**Booklet 4**	**Booklet 5**
Ten-strategy	Addends-compare-strategy	Baseline	Addends-compare-strategy	Ten-strategy	Baseline
Baseline										
Inversion				Inversion		

Students were instructed to solve the problems as quickly and as correctly as possible. The time for each booklet was fixed and we counted the number of problems solved and errors. Students were additionally informed that it would be almost impossible to solve all problems during the period of time given for each booklet. As dependent measure we calculated the average time per problem on addends-compare booklets as compared to baseline booklets.

### Results

**Figure 4:**
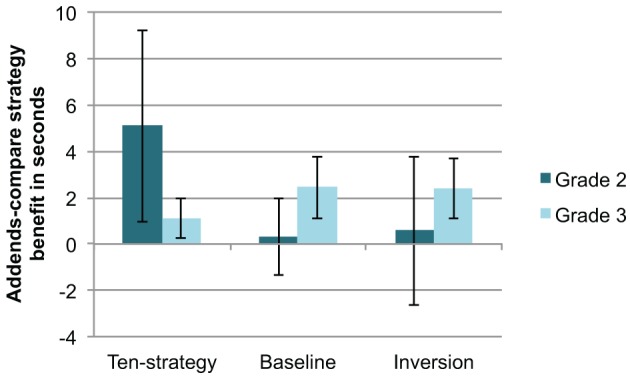
**The mean benefit in seconds of booklets allowing for the addends-compare strategy compared to baseline booklets for the three different warm-up conditions (ten-strategy, baseline and inversion) for the second grade (dark gray) and the third grade (light gray) in Experiment 2**. The error bar displays the 95% confidence interval of the comparison with zero benefit.

After the short warm-up phase, children were still rather slow in calculating the first set of addends-compare booklets and between students variability was rather high (see Table 3). On closer examination, we found that the practice effects were stronger than the effect of problem type. For further analysis we focused on the second addends-compare booklet. We first analyzed the effects of our different warm-up phases on the addends-compare problems. For calculating the addends-compare benefit in second graders, we subtracted for each child the average solution time per problem in Booklet 3 (addends-compare strategy) from the average time per problem in Booklet 2 (baseline). The benefits are depicted in Figure 4 separately for each of the three conditions in second and third graders. In addition, Table 3 presents the average time per problem for every booklet for the second and third grade.

**Table 3:** **Mean time per problem and standard deviation analyzed for booklet type and grade in Experiment 2**.

**Grade**	**Condition**	**Warm-up**	**Booklet 1:**	**Booklet 2:**	**Booklet 3:**	**Benefit**	**Booklet 4:**	**Booklet 5:**
			**Addends-compare**	**Baseline**	**Addends-compare**	**(baseline—addends-**	**Same as**	**Baseline (2)**
			**strategy (1)**		**strategy (2)**	**compare strategy (2))**	warm-up*	
2	Ten-strategy	26.4 (26.8)	28.1 (34.6)	25.2 (23.0)	20.1 (12.0)	5.1 (14.3)	21.9 (22.7)	18.4 (9.2)
	Baseline	23.8 (15.2)	28.3 (24.3)	22.9 (13.4)	22.6 (14.6)	0.3 (5.7)	23.0 (18.4)	22.0 (18.3)
	Inversion	26.3 (29.0)	28.2 (20.4)	25.0 (19.6)	24.4 (16.6)	0.6 (10.7)	17.8 (24.9)	24.4 (21.7)
3	Ten-strategy	10.4 (3.9)	13.2 (3.3)	13.5 (3.7)	12.4 (3.6)	1.1 (2.7)	11.1 (4.6)	12.3 (5.6)
	Baseline	13.9 (7.4)	14.6 (4.5)	15.8 (5.8)	13.3 (2.9)	2.4 (4.2)	13.2 (7.3)	13.4 (5.5)
	Inversion	12.3 (10.3)	15.6 (8.1)	15.8 (6.3)	13.4 (4.5)	2.4 (3.9)	5.9 (5.7)	13.1 (8.2)

^*^See Table 2.

For the second graders with the ten-strategy warm-up phase, we observed a significant benefit on the addends-compare strategy problems compared to baseline problems *t*_(47)_ = 2.48, *p* = 0.05. Second graders with the warm-up problems not allowing for any shortcut did not benefit from the addends-compare booklets relative to the baseline booklets. The inversion problems group also did not show such a benefit either. Third graders, however, seemed to use the addends-compare strategy in every warm-up condition. Each of the three warm-up groups significantly benefitted from the addends-compare strategy [ten-strategy: *t*_(40)_ = 2.64, *p* = 0.05; baseline: *t*_(39)_ = 3.71, *p* = 0.001; inversion: *t*_(38)_ = 3.79, *p* = 0.001]. The time used to solve the addends-compare strategy problems was shorter than that needed to calculate the baseline problems.

We calculated a 2 (problem type: baseline vs. addends-compare booklet) × 3 (warm-up condition: ten-strategy vs. baseline vs. inversion warm-up) × 2 (grade: second vs. third grade) mixed ANOVA with mean benefit time as dependent variable. This ANOVA yield significant main effects of problem type [*F*_(1, 256)_ = 14.98, *p* < 0.001, η^2^_p_ = 0.055] and grade [*F*_(1, 256)_ = 38.44, *p* < 0.001, η^2^_p_ = 0.131] and a significant three-way interaction of problem type × warm-up condition × grade [*F*_(2, 256)_ = 3.75, *p* = 0.05, η^2^_p_ = 0.028]. We found neither a significant main effect for warm-up condition, nor other interaction effects (see Table 4). The three-way interaction suggests that the different warm-up phases differentially affected second and third graders. Whereas the ten-strategy warm-up increased the probability of applying the addends-compare strategy in second graders, it did not in third graders. The results suggest that shortcut to shortcut transfer specific to the underlying mathematical principle was observed in second graders. Third graders, on the other hand, maybe spontaneously used the addends-compare shortcut anyways and thus did not profit from a prior task with a conceptually related shortcut.

**Table 4:** **Experiment 2: Results of the ANOVA problem type × grade × condition**.

		***F***	***p***	**η^2^_***p***_**
Main effect:	Problem type (addends-compare strategy vs. baseline)	14.98	0.00	0.06
	Grade	38.44	0.00	0.13
	Warm-up condition	0.49	0.61	0.00
Inter action:	Problem type (addends-compare strategy) × grade	0.00	0.96	0.00
	Problem type (addends-compare strategy) × warm-up condition	1.57	0.21	0.01
	Warm-up condition × grade	0.14	0.87	0.00
	Problem type (addends-compare strategy) × warm-up condition × grade	3.75	0.02	0.03

One could argue that second graders did not show transfer from an inversion warm-up to addends-compare problems, because they did not discover the shortcut option in the inversion problems. Our manipulation checks do not support this alternative explanation. We analyzed the Booklets 4 and 5 (induction shortcut—and respective baseline). The results suggested that students were capable of using the inversion strategy (see Table 5). For the second graders, a 2 (Booklet 4 vs. 5) × 3 (warm-up condition) ANOVA revealed a significant interaction effect of both factors, [*F*_(2, 139)_ = 3.20, *p* = 0.05, η^2^_p_ = 0.044]. It depended on the warm-up condition, whether the shortcut in Booklet 4 was used.

**Table 5:** **Results of the ANOVA problem type × condition separately for grade 2 and 3**.

		**Grade 2**			**Grade 3**		
		***F***	***p***	η^2^_***p***_	***F***	***p***	η^2^_***p***_
Main effect:	Problem type (addends-compare strategy)	4.92	0.03	0.03	35.04	0.00	0.23
	Warm-up condition	0.23	0.80	0.00	1.95	0.15	0.03
Inter action:	Problem type (addends-compare strategy) × warm-up condition	2.97	0.06	0.04	1.73	0.18	0.03

For the third graders we also found an interaction effect of Booklet 4 vs. 5 and warm-up condition, [F_(2, 117)_ = 15.41; *p* < 0.001, η^2^_p_ = 0.208]. While there was a pronounced inversion effect, surprisingly, neither baseline warm-up condition nor the ten-strategy warm-up condition showed a ten-strategy effect in the booklets administered at the end of the experiment. We did not find relevant effects when repeating the above analyses with error rate as dependent variable, but needless to say we found different error rates in grade two and three (see Supplementary materials).

### Discussion

In Experiment 2, we tested whether it is possible to make students to spot and apply a shortcut strategy by first providing an easy-to-find shortcut strategy based on the same mathematical principle vs. one based on a different principle. Our findings suggest that in second graders, transfer was related to the mathematical principle rather than to general motivational factors. There was no indication that second graders were motivated to search for and apply *any* shortcuts after being offered the first one. If the additional conceptual link between the two different strategies is the reason for the transfer, this would support understanding of adaptive expertise as the ability to apply meaningfully learned procedures flexibly and creatively (Hatano and Oura, 2003). The inversion warm-up phase—an easy-to-find shortcut that is not based on commutativity—did not lead to increased usage of the addends-compare strategy. While inversion did not promote transfer, our manipulation check suggested that inversion was indeed used. This is in line with Robinson and Dubé (2009) who found that the inversion shortcut is easier to apply than associativity (which is similar to commutativity). In both studies (Robinson and Dubé, 2009; Dubé and Robinson, 2010), inversion shortcut use was far more frequent than the associativity-based strategy. Focusing on commutativity as model case a limitation of the experiment is that we so far only used one shortcut not based on commutativity (i.e., inversion) in order to differentiate between transfer effects based on motivation vs. on mathematical principles shared by subsequently offered shortcut options. For instance, it would be interesting to know whether the current setup can be turned around with inversion usage as dependent variable and commutativity vs. inversion warm-up as independent variable (cf. Dowker, 2014). Generalizability beyond the specific pairing of shortcuts tested here might for instance depend upon the relative difficulty of shortcuts used as warm-up and dependent variable.

While the results suggest that second graders profited from shortcut-to-shortcut transfer based on commutativity, third graders did not seem to benefit from such extra scaffolding. Spontaneous usage of the addends-compare strategy was not improved further by a warm-up condition with a shortcut-option based on the same mathematical principle. We assume that in this age group, the concept of commutativity is more developed so that extra support is less needed. With further experience, students become increasingly able to rapidly generate adequate actions with less and less effort (Ericsson, 2008). In line with these findings, differences between second and third graders in their mathematical abilities are mirrored in functional changes of the brain. Rosenberg-Lee et al. (2011) examined the behavioral and neurodevelopmental changes between grades 2 and 3 and found that arithmetic complexity was associated with regions implicated in domain-general cognitive control but also regions for numerical arithmetic processing. The results showed that brain response and connectivity relating to an arithmetic task significantly change within the narrow 1-year interval.

## General discussion

We presume that one crucial feature of expertise is the ability to spontaneously recognize where and when knowledge can be applied to simplify task processing. In some domains, it is necessary for everyday life to develop this ability. Research of expertise showed that experts are more flexible and creative in their thought pattern. For instance, “super experts” were more flexible to find an optimal solution despite distraction by a non-optimal but salient solution of a chess problem (Bilalić et al., 2008a). Players at lower levels of expertise reported that they were looking for a better solution, but their eye movements showed that they continued to look at features related to the solution they had already thought of (Bilalić et al., 2008b). For expertise in object recognition, Harel et al. (2013) developed an interactive framework, which posits that expertise emerges from multiple interactions within and between the visual system and other cognitive systems, such as top-down attention and conceptual memory. The interplay between these other, multiple cognitive processes and perception are often not consciously accessible for the experts themselves (Palmeri et al., 2004).

In some parts of arithmetic, procedural and conceptual knowledge start to develop even before primary school. In the first years of primary school, integration of different fragments of procedural and conceptual knowledge should lead to a knowledge base that allows to spontaneously spot and apply shortcut options already in primary school. If successful, knowledge integration should lead to transfer between procedurally different shortcuts that are based on the same mathematical principle and therefore likely are both associated to the respective conceptual knowledge. For the case of commutativity, we tested whether different strategies that are based on the same principle trigger each other via the concept and so could support flexibility in strategy use. According to the adaptive expertise metaphor (e.g., Hatano, 1988; Star and Rittle-Johnson, 2008; Verschaffel et al., 2009) children first of all need to spontaneously recognize where knowledge can be applied.

Experiment 1 provided first evidence that children who are provided an opportunity to spontaneously spot and apply one shortcut might be more inclined to search for and use a second shortcut, once the first one no longer applies. This is in line with the suggestion to differentiate between (a) quick and accurate routine-based solving from (b) an adaptive use of solution strategies, which draws upon conceptual understanding (Hatano, 1988). Experiment 2 verified that transfer occurred from one shortcut to another. It furthermore specified that this transfer effect was not only based on motivation. While we obtained transfer (at least in second graders) from one commutativity-based shortcut to another commutativity-based shortcut, no transfer was observed between inversion and commutativity. Thus, our results are in line with the view that links between different elements of procedural knowledge and potentially conceptual knowledge (compare Haider et al., 2014) are used to spontaneously spot and apply shortcut options.

Several studies on commutativity have shown that children have at least some understanding of the concept of commutativity before entering school (Siegler, 1989; Resnick, 1992; Cowan and Renton, 1996; Wilkins et al., 2001; Canobi et al., 2003) and already first graders seem to understand the commutativity principle (Canobi et al., 2002). We thus focused on triggering the usage of knowledge rather than knowledge acquisition as such. In primary school, children should link different strategies based on the same concept and develop the ability to select an efficient strategy for the current problem (Verschaffel et al., 2009). As implied by these authors in the adaptive expertise metaphor, the learner should be able to spot and apply options for a shortcut independently without having to rely on instruction or explicit cues. In a similar vein, research on skill acquisition and expertise stresses the importance of linking perceptual skills and principle-knowledge in order to be able to spontaneously spot and apply shortcuts (e.g., Gentner and Toupin, 1986; Koedinger and Anderson, 1990; Haider and Frensch, 1996; Anderson and Schunn, 2000; Bilalić et al., 2008a; Frensch and Haider, 2008). Adaptive strategy use can be regarded as the ability to select procedures that can simplify the solution of a problem (Selter, 2009). In the end the person should be faster and/or the solution should be more accurate. Strategy use can be seen as an indicator for the state of development of a mathematical concept. Adaptive strategy use necessitates shifts between: (a) calculating problems in the general mode (b) investing some time and effort to search for shortcut options, and (c) using a shortcut option. We are interested in factors that can tip the balance on the exploitation-exploration continuum. Experts know when to search for a new shortcut strategy and when not, children have to learn how much time and effort they want to spend for searching. Teachers etc. cannot sustainably take over the regulation of this dilemma calculating in standard way or flexible change strategies—they can only help children to calibrate the balance between flexibility vs. stability (or exploration vs. exploitation).

We have to acknowledge that the effects of spontaneously using a shortcut were small in many cases of the current experiments and the variability across students was large. This is to be expected when taking into account the difference between competence and performance (i.e., principle knowledge and application). Larger estimates of both procedural and conceptual knowledge have been obtained when knowledge was probed more directly (Prather and Alibali, 2009). Direct probing, however, does convey to the students that and which shortcut options exist. It is therefore not suitable when trying to measure the extent to which knowledge about a mathematical principle is applied spontaneously (cf. Haider et al., 2014). In addition, Robinson and Dubé (2012) have suggested that personality characteristics bridge between knowledge and application. They argued that some children have more positive attitudes toward accepting strategies that are highly efficient but are novel to their current strategy repertoire of algorithmic approaches. In a similar vein, (Guerrero and Palomaa, 2012) highlighted that some children change their strategies during calculation while some do not. Furthermore, children change their strategies for different reasons. It is not always the goal to choose the most efficient strategy (Newton et al., 2010) suggested that flexibility involves the use of strategies, which are considered the most appropriate for a given problem. They also discussed what “appropriate” means. It could be the most efficient or the most understandable strategy in a given situation. Which strategy in general is used depends on the problem, the numbers presented and other contextual, developmental, or personal factors (Newton et al., 2010; Guerrero and Palomaa, 2012). An U-shaped relationship between knowledge/understanding and variety of strategy use suggests that novices as well as experts may use a large variety of strategies (Siegler and Jenkins, 1989; Dowker et al., 1996). Experts like mathematic students used large numbers of appropriate strategies (Dowker et al., 1996) whereas children (novices) may use a large variety of appropriate and inappropriate strategies, because they have not yet acquired a small set of well-learned strategies (Dowker et al., 1996). In contrast to this assumption Newton et al. (2010) argued that low achieving students might be particularly appreciative and excited about a focus on multiple strategies to compare the possible ways to solve the problem and maximize the accuracy. Although the idea is prominent that an educational approach for low achieving children should promote routine mastery of a single well-thought solution strategy for a given type of problems (e.g.,Woodward and Baxter, 1997; Baxter et al., 2001). Future work should explore how students at different ability levels profit from sequences of problems allowing for different shortcuts based on the same mathematical principle.

In order to optimize the chances to measure spontaneous (i.e., no cues and no instruction) recruitment of knowledge about the commutativity principle we chose a paper-and-pencil test in the classroom in Experiment 2. Our informal observations suggest that children taking part in an eye tracking study on mental arithmetic appreciate that the measurement is (not only) about whether they solve the problems correctly, but also on how they solve them. The paper-and-pencil method was closer to usual test situations in the classroom. Children focused on being fast and accurate rather than on the fact that someone might be trying to assess *how* they solved the problems. Verschaffel et al. (2009) highlighted the importance of ecological validity for studies on adaptive expertise. We suggest that trial-by-trial process measures (as in our eye tracking experiment) and ecologically valid but less sensitive methods (as in Experiment 2) should be combined to convey the full picture. For instance, eye tracking can help to figure out whether increased time demands after a change in shortcut option reflect prolonged solution times or, alternatively, a mixture of prolonged solution times plus time invested in search for alternative shortcut options. Potentially, learners at different levels of expertise might differ in both the efficiency in spotting shortcuts as well as in using them. For instance, third graders might have discovered the options for the addends-compare shortcut relatively quickly even without a fitting warm-up condition.

In line with the research on adaptive expertise Verschaffel et al. (2009) or Star and Rittle-Johnson (2008) defined flexibility in problem solving as knowledge of multiple strategies and their relative efficiency. In addition to weighing different strategies according to their efficiency, students need to weigh the potential costs and benefits of flexible strategy usage. There are time costs of switching between strategies, once a shortcut option has been discovered (Lemaire and Lecacheur, 2010). Luwel et al. (2009) found longer response times but no reduced accuracy and the size of these switching costs varied as a function of the associative strength between a strategy and a particular problem. More importantly, there is a dilemma between (a) investing time and attention in order to spot potential shortcut options that might or might not exist and (b) using processing strategies readily available (e.g., Jepma and Nieuwenhuis, 2011). Thus, process measures that provide evidence on when, how and to what extent students invest in spotting and applying shortcuts (Haider and Rose, 2007) are necessary in order to better understand the bases of the transfer effect observed in Experiment 2. To illustrate the search process, we additionally used eye tracking assessment in the Experiment 1. On the one hand this is a more specific method than paper pencil and on the other hand we could measure the shift of attention. The eye tracking results are in line with the view of (Robinson and LeFevre, 2012). For discovering new strategies, children need to shift their attention to the relevant part of the problem. The eye movement patterns were different for the different shortcut strategies and fit to the points of interests of the according strategies.

### Conflict of interest statement

The authors declare that the research was conducted in the absence of any commercial or financial relationships that could be construed as a potential conflict of interest.
