# Supplementary material for: Spontaneously spotting and applying shortcuts in arithmetic—a primary school perspective on expertise
Source: Front Psychol. 2014 Jun 10;5:556. doi: 10.3389/fpsyg.2014.00556 (PMC4051128; doi:10.3389/fpsyg.2014.00556)
Supplement: Supplementary file 1 [file DataSheet1.PDF]

Supplementary Material

Sample of the material

Table S1

Examples of the first five problems of each problem type (*ten-strategy problems* and *baseline*) in the parallel sets A and B. The results printed in italics had to be filled in by the participants.

Set A		*Set B*	
Ten-strategy	*Baseline*	*Ten-strategy*	*Baseline*
4+5+6=*15*	4+3+8=*15*	6+5+4=*15*	8+4+3=*15*
3+2+7=*12*	3+5+4=*12*	7+2+3=*12*	3+4+5=*12*
5+6+5=*16*	8+5+3=*16*	5+9+5=*19*	8+3+5=*16*
7+4+3=*14*	2+5+7=*14*	3+4+7=*14*	5+2+7=*14*
2+7+8=*17*	9+3+5=*17*	8+7+2=*17*	5+3+9=*17*
Set A		*Set B*	
Addends-compare	*Baseline*	*Addends-compare*	*Baseline*
3+5+4=*12*	5+3+4=*12*	4+3+5=*12*	4+5+3=*12*
4+9+8=*21*	8+9+4=*21*	5+7+9=*21*	8+4+9=*21*
4+8+9=*21*	6+7+8=*21*	5+9+7=*21*	7+8+6=*21*
6+2+5=*13*	5+2+6=*13*	2+6+5=*13*	2+5+6=*13*
9+7+2=*18*	2+7+9=*18*	4+5+9=*18*	7+9+2=*18*
2+9+7=*18*	9+4+5=*18*	5+4+9=*18*	5+9+4=*18*

Additional analyses of the error rates

Experiment 1

After analyzing the error rates for both strategies (ten-strategy and addends-compare strategy) we compare differences in the % error rates with a T-Tests between the baseline and addends-compare booklet (Table A3). The results showed no significant differences.

Experiment 2

We found no significant differences in the % error rates in T-Tests between the baseline and addends-compare booklet (Table A2). Also a 2 (problem type: baseline vs. addends-compare booklet) X 3 (warm-up condition: ten-strategy vs. baseline vs. inversion warm-up) X 2 (grade: second vs. third grade) ANOVA showed no significant effects for the error rate except the significant main effect for grade $F(1, 256)=16.63$, $p<.00$, $\eta p^2=.06$.

Table S2

Error rates per problem type and grade (Experiment 1)

	% errors		
		Baseline	*p*
Addends-compare	27.02	30.15	.50
Ten-strategy	15.03		

Table S3

Error rates per problem type and grad (Experiment 2)

		% errors		
		Baseline	Addends-compare	*p*
Grade 2	ten-strategy	16.89	19.43	.22
	baseline	19.42	20.14	.79
	inversion	20.10	18.19	.52
Grade 3	ten-strategy	6.62	7.80	.56
	baseline	12.66	10.34	.53
	inversion	8.96	12.50	.11
